# Chorea Hyperglycemia Basal Ganglia Syndrome in a 63-Year-Old Male

**DOI:** 10.1155/2018/9101207

**Published:** 2018-11-05

**Authors:** Michael Sperling, Roshan Bhowansingh

**Affiliations:** ^1^Carilion Clinic Virginia Tech School of Medicine, Internal Medicine, 1906 Belleview Avenue, Roanoke, VA 24014, USA; ^2^U.S. Department of Veteran Affairs, Internal Medicine, 1970 Roanoke Blvd., Salem, VA 24153, USA

## Abstract

Chorea hyperglycemia basal ganglia syndrome (CHBG) is a rare condition that manifests within the setting of uncontrolled nonketotic diabetes mellitus. The objective of this case report is to present a patient found to have CHBG and provide a timeline in terms of his workup and subsequent treatment. We also present a commentary on the current understanding of the pathophysiology and treatment and how this was applied to our patient. The case involves a 63-year-old poorly controlled diabetic male who presented with a one-week history of uncontrolled choreiform movements of his left upper extremity. His initial glucose level was 339 mg/dl. HbA1C was 9.9%. CT scan of the head demonstrated an abnormal increased intensity within the right lenticular nucleus and right caudate head most likely due to microcalcifications/mineralization. MRI of the brain demonstrated nonspecific T1 and T2 hyperintense abnormalities in the same area about the right basal ganglia. These findings were consistent with the movement pattern he was displaying and with a diagnosis of CHBG. Gradual control of his blood sugar levels over 48 hours led to resolution of his choreiform symptoms. After better medication adherence as an outpatient, endocrinology follow-up 6 months after discharge found his HbA1C drop to a level of 7.1%. There was no recurrence of his symptoms. CHBG is a rare manifestation of poorly controlled diabetes but is the one that clinicians should be aware of. Early recognition and gradual treatment of elevated blood glucose levels appear to lead to total resolution of choreiform symptoms.

## 1. Syndrome

Chorea hyperglycemia basal ganglia syndrome (CHBG) is a rare condition that manifests in the setting of uncontrolled nonketotic diabetes mellitus. It is best characterized by the manifestation of hemichorea-hemiballism with uncontrolled blood sugar levels. Not much is currently known regarding the pathogenesis of this unique and underrecognized condition. An extensive literature review revealed several proposed theories. Most of these theories revolve around the recognition that hyperglycemia may impair the cerebral autoregulatory mechanisms of the central nervous system. This can lead to hypoperfusion and sequential activation of anaerobic metabolism [1]. This results in the depletion of gamma-aminobutyric acid (GABA) within the basal ganglia neurons [1]. GABA and acetate are depleted rapidly in nonketotic hyperglycemia which causes a reduction in acetylcholine synthesis [2]. The hyperviscosity induced by hyperglycemia then causes a disruption of the blood-brain barrier and a resulting transient ischemia of the vulnerable striatal neurons [3]. The synergistic effects of uncontrolled hyperglycemia and vascular insufficiency are believed to cause an incomplete transient dysfunction of the striatum, which consequently leads to hemichorea-hemiballism within these patients [4]. Histological findings in patients with CHBG syndrome have been reported to be characterized by selective neuronal loss, gliosis, and reactive astrocytes without any evidence of hemorrhage or infarction within the striatal areas [4].

There are a limited number of case reports and articles regarding CHBG. Several case reports have documented that hemichorea-hemiballism can occur a few weeks after the blood glucose levels are controlled and actively being treated. This suggests a delayed reaction to the elevated blood sugar levels. Patients have been known to present with acute-onset hemichorea in the setting of well-controlled sugars with an elevated HbA1C or in severely hyperglycemic states. Chorea is characterized by involuntary and irregular movements that are not rhythmic or repetitive. Hemichorea is the occurrence of these movements on one side of the body [5]. When a patient presents with symptoms such as these, the differential typically involves hemorrhagic or ischemic stroke, neoplasm, systemic lupus erythematosus, Wilson's disease, or thyrotoxicosis [6]. CHBG is usually not a top consideration. One such case of CHBG presented in an elderly female with type II diabetes and right-sided hemichorea of acute onset during an episode of nonketotic hyperglycemia. Her MRI revealed a characteristic hyperintensity and T1 hyperintensity in the left basal ganglia [7]. Another such report described a patient from Saudi Arabia with left-sided hemichorea and a history of longstanding uncontrolled diabetes. Imaging showed unilateral right striatal hyperintense signal changes in a T1-weighted MRI [8]. These unique MRI findings are typically present in patients who present with CHBG and have been described by radiologists as consistent with hemichorea-hemiballism associated with nonketotic hyperglycemia in primary diabetes mellitus [9]. We present the case of a gentleman that presented to the ER with acute onset of hemichorea in the setting of poorly controlled diabetes mellitus which was eventually determined to be CHBG.

## 2. Case

**Figure 1:**
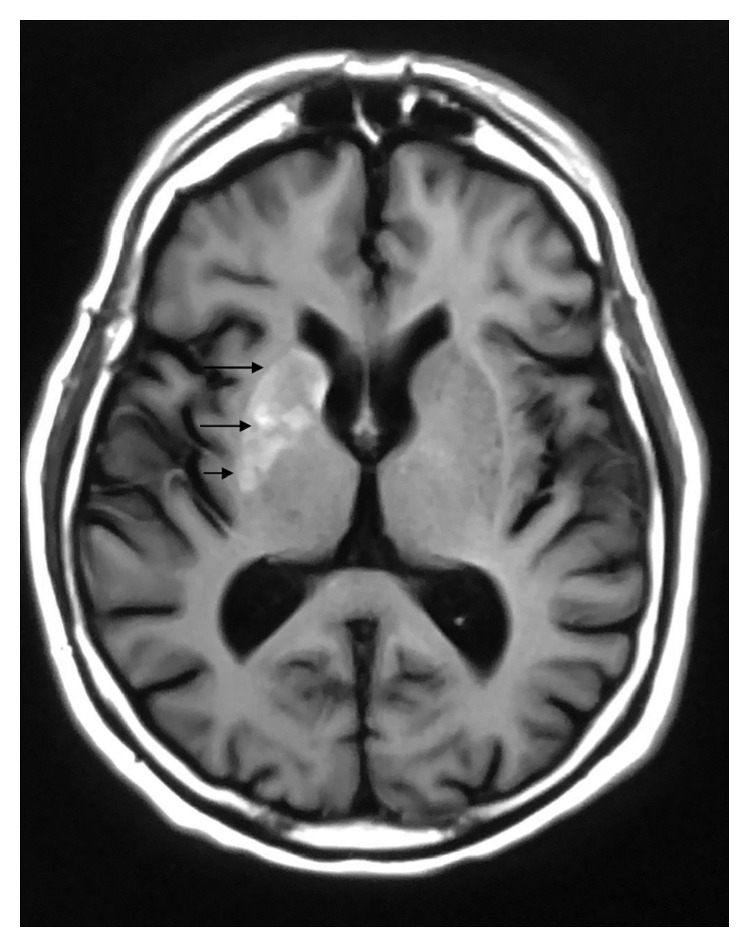
MRI brain precontrast T1-weighted axial spin echo image. Arrows highlight the right basal ganglia showing abnormal hyperintense signal.

**Figure 2:**
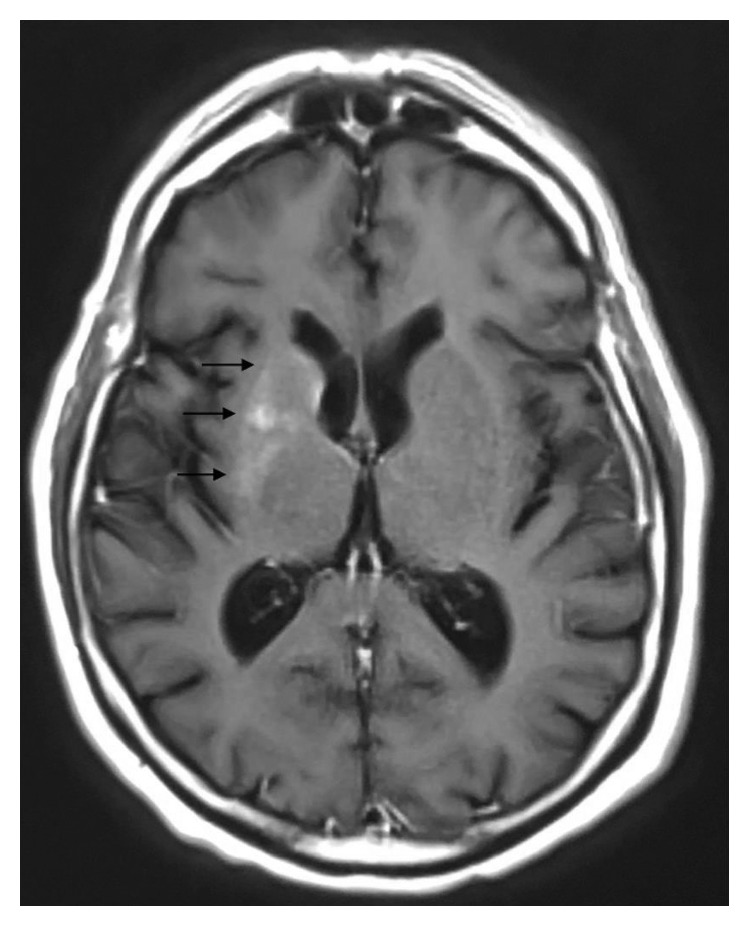
MRI brain postcontrast T1-weighted axial spin echo image. Arrows highlight hyperintense signal abnormalities in the right basal ganglia with no change in enhancement postcontrast.

**Figure 3:**
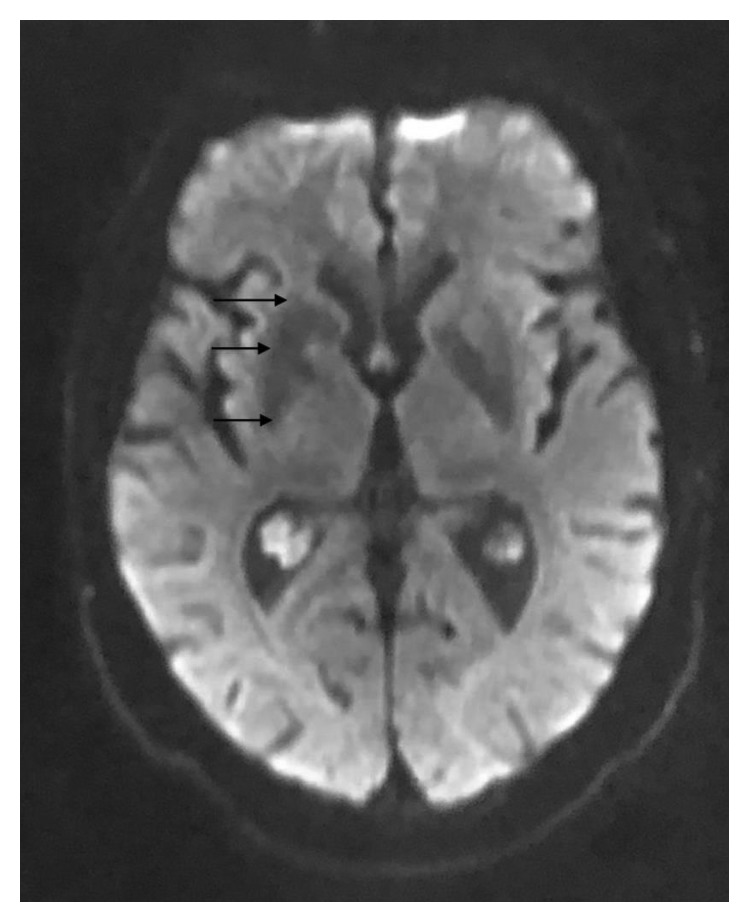
MRI brain with diffusion-weighted axial image. Arrows highlight the corresponding right basal ganglia with no hyperintense signal abnormality to suggest ischemia.

A 63-year-old Caucasian male presented with a one-week history of uncontrolled choreiform movements of his left upper extremity. As described by the patient, his left arm began “jerking uncontrollably out of nowhere” while working in his shed at home. Prior to this development, the patient stated he had one similar episode a year ago which lasted for two days and resolved spontaneously. He did not seek evaluation at that time. His past medical history consisted of uncontrolled insulin-dependent diabetes, hypertension, schizoaffective disorder, and polysubstance abuse in remission. He reported having diabetes mellitus for at least 10 years. Reviewing the medical records, his previous HbA1C readings ranged from 13.8% to 12.4% over a 12-month span prior to his admission. His average blood sugar readings over this time ranged from 300 to 350 mg/dl. BMI was 25.3 kg/m^2^. His home insulin regimen included 10 units of NovoLog three times a day with meals and Lantus 20 units every morning. He was not on any oral diabetic medications. The patient reported that he was not compliant with his home insulin medications. Notably for comparison, an MRI brain performed 5 months before for a fall did not reveal any abnormalities. He denied any recent medication changes, illnesses, or headaches. His ESR and CRP were elevated, and his initial glucose level was 339 mg/dl. HbA1C was 9.9% on the day of his admission. Vital signs were normal upon presentation, and physical exam was benign aside from the hemichorea movements of the left upper extremity. Our differential diagnosis included neoplastic disorders (metastatic brain disease and brain tumor), Huntington's disease, ischemic or hemorrhagic stroke, trauma, and drug or chemical toxicity (dopamine agonist or phenytoin). CT scan of the head showed areas of high density in the right lenticular nucleus and right caudate head. Subsequent precontrast MRI demonstrated T1 and T2 hyperintense abnormalities in the caudate nucleus portion of the right basal ganglia (Figure 1). Postcontrast MRI of the brain showed no abnormal enhancement excluding the possibility of a mass lesion (Figure 2). Diffusion-weighted MRI brain images did not reveal any abnormal restricted diffusion in the right basal ganglia which excluded ischemia (Figure 3). All of these changes were consistent with the movement pattern he was displaying and with a diagnosis of CHBG. Other conditions that have been known to cause hyperintense imaging abnormalities on MRI such as this include various cellular respiratory toxins such as carbon monoxide, methanol, and cyanide. Leigh disease and hyperammonemia from chronic cirrhosis have also been known to cause this. Our patient did not have any history that would correlate with these alternative possibilities, and additionally, none of these alternatives have been known to present with hemichorea. Inpatient treatment consisted of restarting the patient's home insulin regimen with the addition of inpatient corrective coverage. This included Lantus 20 units every morning and 10 units of NovoLog 3 times a day with meals. The patient's symptoms eventually resolved with control of his blood sugar levels. Control was achieved over roughly 48 hours during which our patient's blood glucose levels dropped from an average of mid-300s to the 170–200 mg/dl range. The choreoathetosis progressed from continuous movements to intermittent and then finally to resolution.

Endocrinology was consulted for assistance with the patient's difficult glycemic control. During his posthospitalization outpatient course, our patient began to consistently adhere to his outpatient diabetic medications. Lantus was increased to 40 units every morning and NovoLog was increased 13 units 3 times a day with meals. His HbA1C 6 months after discharge had improved to 7.1%. There were no further occurrences of choreiform movements. This time course of events demonstrates how treatment of hyperglycemia can adequately treat and lead to complete resolution of hemichorea-hemiballismic movements in patients with CHBG. In this case, symptomatic improvement began being observed 48 hours after initiation of glucose control.

## 3. Discussion and Conclusions

CHBG is a true rarity and deserves awareness in light of the fact that it is likely underdiagnosed in the western population [10]. Most patients presenting with CHBG are of older age, female sex, and Asian origin [7]. CHBG has also been described as more commonly involving the upper extremities as opposed to the lower extremities; however, some cases of lower extremity involvement have been reported [11].

CHBG should be considered when characteristic T1 and T2 hyperintense abnormalities are observed on MRI, and there is hemichorea-hemiballism with a history of uncontrolled diabetes mellitus. Based on the available literature, the majority of patients with CHBG syndrome have a benign clinical course that can be managed medically. The onset of the disorder usually coincides with severe hyperglycemia with a chronological relationship between restoration of blood glycemic levels and improvement of the chorea [11]. Thus, the mainstay of treatment appears to be gradual glycemic control leading to either a partial or complete resolution of hemichorea-hemiballism [12]. The importance of avoiding hypoglycemia has been well established and should also be taken into consideration. According to the literature, there is no clear consensus on how quick blood sugar control should be achieved in CHBG. A gradual improvement in blood glucose over a period of several days seems to be appropriate and leads to improvement of the choreiform movements while avoiding hypoglycemia. The clinical and radiological signs have been reported to take about 6 months to resolve after the correction of hyperglycemia [12]. Once again, the majority of case reports in the current medical literature have demonstrated that most patients have complete recovery from their hemichorea-hemiballism after adequate control of hyperglycemia. This demonstrates that CHBG once recognized and treated is a disorder with a good prognosis and is the one that clinicians should be aware of [11].
